# Noise exposure during prehospital emergency physicians work on Mobile Emergency Care Units and Helicopter Emergency Medical Services

**DOI:** 10.1186/s13049-017-0459-9

**Published:** 2017-12-06

**Authors:** Mads Christian Tofte Hansen, Jesper Hvass Schmidt, Anne C. Brøchner, Jakob Kjersgaard Johansen, Stine Zwisler, Søren Mikkelsen

**Affiliations:** 1 Department of Anesthesiology and Intensive Care, Odense University Hospital, Odense C, 5000 DK Denmark; 2 Department of Audiology, Odense University Hospital, Odense C, 5000 DK Denmark; 3 Department of ENT, Head and Neck Surgery, Odense University Hospital, Odense C, 5000 DK Denmark; 4 Institute of Clinical Research, University of Southern Denmark, Odense M, 5230 DK Denmark; 5 National Helicopter Emergency Medical Services, Pre-hospital Center Region of Central Denmark, Aarhus C, 8200 DK Denmark; 6 Mobile Emergency Care Unit, Odense University Hospital, Odense C, 5000 DK Denmark

**Keywords:** Noise exposure, Pre-hospital, Ambulance, Helicopter, HEMS, Occupational guidelines, Mobile Emergency Care Unit

## Abstract

**Background:**

Prehospital personnel are at risk of occupational hearing loss due to high noise exposure. The aim of the study was to establish an overview of noise exposure during emergency responses in Mobile Emergency Care Units (MECU), ambulances and Helicopter Emergency Medical Services (HEMS). A second objective was to identify any occupational hearing loss amongst prehospital personnel.

**Methods:**

Noise exposure during work in the MECU and HEMS was measured using miniature microphones worn laterally to the auditory canals or within the earmuffs of the helmet. All recorded sounds were analysed in proportion to a known tone of 94 dB. Before and after episodes of noise exposure, the physicians underwent a hearing test indicating whether the noise had had any impact on the function of the outer sensory hair cells. This was accomplished by measuring the amplitude level shifts of the Distortion Product Otoacoustic Emissions. Furthermore, the prehospital personnels’ hearing was investigated using pure-tone audiometry to reveal any occupational hearing loss. All prehospital personnel were compared to ten in-hospital controls.

**Results:**

Our results indicate high-noise exposure levels of ≥80 dB(A) during use of sirens on the MECU and during HEMS operations compared to in-hospital controls (70 dB(A)). We measured an exposure up to ≥90 dB(A) under the helmet for HEMS crew. No occupational hearing loss was identified with audiometry. A significant level shift of the Distortion Product Otoacoustic Emissions at 4 kHz for HEMS crew compared to MECU physicians was found indicating that noise affected the outer hair cell function of the inner ear, thus potentially reducing the hearing ability of the HEMS crew.

**Discussion:**

Further initiatives to prevent noise exposure should be taken, such as active noise reduction or custom-made in-ear protection with communication system for HEMS personnel. Furthermore, better insulation of MECU and ambulances is warranted.

**Conclusion:**

We found that the exposure levels exceeded the recommendations described in the European Regulative for Noise, which requires further protective initiatives. Although no hearing loss was demonstrated in the personnel of the ground-based units, a reduced function of the outer sensory hair cells was found in the HEMS group following missions.

## Background

In recent years, the topic of occupational noise exposure has received increased attention. The risk of permanent hearing loss due to work-related noise exposure is a well-known issue with an estimated 22 million U.S. workers exposed to hazardous noise each year [1]. Moreover, 34% of those exposed to hazardous noise report no use of ear-protective gear [1]. As stated in the E.U. Directive 2003/10/EC for Noise Exposure, employees should not be exposed to more than 87 dB(A) on a daily basis (during 8 h) [2]. In this directive, it is recommended that hearing protective gear should be available to employees exposed to noise levels of 80 dB(A), and it is stated that its use should be mandatory at values exceeding 85 dB(A) [2]. It is well known that emergency responses in themselves pose a risk to the pre-hospital personnel in the form of traffic accidents [3]. Another occupational risk that in particular may be relevant to emergency medical technicians (EMTs) and pre-hospital physicians working in Mobile Emergency Care Units (MECU) or Helicopter Emergency Medical Services (HEMS) is noise exposure. Physicians and EMTs may not be fully aware of the intensities of noise to which they are exposed during the day, with a large range of noisy tasks performed in the pre-hospital environment. Thus, they may not be aware of the possible consequences of this exposure on their hearing ability.

A few studies have evaluated patient and crew noise exposure during ambulance transport both in ground-based units, in fixed-wing operations (medical aeroplanes) and in alpine rescue helicopters with contradictive findings [4–7]. According to one study, transportation by fixed-wing airplane does not affect hearing with noise exposure levels reported just below the limit for acceptable occupational noise exposure (85 dB(A)). However, in that particular study, no hearing tests were carried out, and the question of possible hearing loss was thus unresolved [4]. Reducing noise exposure with protective gear (up to 10 dB) to the flight crew is already a priority and contributes to an acceptable equivalent noise level below the 85 dB(A) threshold [4]. However, noise exposure during alpine helicopter rescue operations seems to exceed the national occupational levels with exposures equivalent to noise levels of >85 dB(A) recorded during 2726 missions from four bases [7]. The setting during an alpine rescue differs from other Helicopter Emergency Medical Services by often “hot-loading” the patient (the patient is loaded while the engine is on). This principle, however, is also used on occasion by non-alpine HEMS rendering the ensuing noise exposure relevant to these organisations. Protective gear is already used but may be insufficient in protecting the crew from loss of hearing [7], especially when working outside of the helicopter. A study in 521 French military helicopter pilots who underwent pure-tone audiometry prior to their annual medical examination exhibited abnormal hearing especially in the high frequencies at 3 kHz and 6 kHz [8]. Moreover, fighter- and helicopter pilots seemed more vulnerable to hearing loss than transport pilots [8]. Thus, a risk of work-related hearing has been established in the flying services.

Emergency medical technicians (EMTs) operating in ground ambulances seem to be exposed to high levels of noise [5, 6]. The highest recorded sound levels have been measured with sirens engaged en route during live emergency responses. One study showed that when the sirens were on, all noise values measured inside the cabin exceeded the national occupational health regulation of 85 dB(A) as a mean of 96 dB(A) was measured [5]. The average siren noise exposure during a work day of 8 h was 16.22 min (range 5–33 min), reaching 20.33 min on busy days (range 7–42 min) [5]. These measurements were only conducted in four ambulances under three different circumstances (12 recordings in total) [5]. Based on 31 noise recordings, a second study found noise levels up to 84 dB(A) in the front passenger seat [6]. The two studies concluded that the occupational noise exposure resulted in excessive hearing loss among EMTs especially in the high frequencies, which typically are the frequencies involved in noise-induced hearing loss [5, 6]. Additionally, they reported a noise level ranging between 93 and 106 dB(A) inside the cabin during the use of sirens [5] which, according to E.U. directive, would require the use of ear-protective gear as the noise level exceeds 85 dB(A) [2]. Excessive noise exposure may lead to a temporary threshold shift (TTS) in hearing. This TTS is normally quantified with audiometry but the level shift of amplitudes measured with Distortion Product Otoacoustic Emissions (DPOAE) can be used as a measure of altered sensory outer hair cell (OHC) function in the inner ear which can be an indicator of affected hearing [9]. In that study, a temporary shift in pure-tone audiometric thresholds and a reduction of the DPOAE amplitudes were documented within the frequency range 1–6 kHz following exposure to excessive noise at sporting events [9]. The measurement of sensory OHC function can be performed quickly with DPOAE measurements making it feasible in the pre-hospital environment for detecting impacts on hearing [9].

The aim of this study was to investigate the overall noise exposure during pre-hospital physicians’ pre-hospital work in the MECU and civilian HEMS and to clarify whether a change in amplitude levels of DPOAE occurs following noise exposure. Any association between noise exposure from MECU and HEMS and change in DPOAE amplitudes was investigated. Furthermore, in an attempt to reveal any occupationally related hearing loss, a group of EMTs, pre-hospital physicians and in-hospital controls underwent a standardised pure-tone audiometry.

## Methods

### Setting and study population - MECU

Measurements of noise exposure were made during emergency responses with the Danish ground-based MECUs of Odense, Kolding and Aarhus in the period of March 2016 to August 2016. The MECUs consist of rapid-response cars operating all year round. The MECUs are manned with an anesthesiologist and an EMT with special training. The MECUs operate as part of a three-tiered system in which the MECU supplements the ordinary ambulance manned with EMTs and/or paramedics. Following treatment, the MECU may finalize treatment at the scene, admit the patient to hospital without physician escort, or escort the patient to the hospital. Each day, the three MECUs are dispatched to 13, 10 and 15 emergencies on average. Odense and Aarhus both use a custom-made Mercedes ML350 while the MECU in Kolding uses a custom-made BMW X3 xDrive20d. All vehicles have top speeds of approximately 230 km/h. The Danish law has specific requirements regarding sirens and emergency lights with which all three MECUs comply. The siren speaker is a dual speaker placed in the front bumper of all cars. In total, 28 physicians from the three MECUs were enrolled in the study.

### Setting and study population - HEMS

An identical approach was used to investigate the effect of noise exposure on the pre-hospital anesthesiologists and pilots in the Danish national HEMS in the same period. The national HEMS consist of three helicopters stationed in three geographically different places in Denmark, ensuring the optimal coverage of the country. The HEMS of Ringsted serves approximately 2.0 million citizens and has 3 daily missions on average. The dispatch criteria for the HEMS are tighter than those of the MECUs as they are only dispatched to emergencies with life-threatening conditions [10]. The helicopter is an Airbus EC135 P2e helicopter with an average ground speed of 240 km/h. It is a small helicopter suitable for take-off and landing in both urban and rural areas. The HEMS is manned by a pilot, an anesthesiologist and a HEMS Crew Member (HCM). The helicopter generates noise inside the cabin in the range of 84–96 dB depending on the task performed (take-off, fly-by or landing) as stated in the Type-Certificate Data Sheet for Noise No. EASA.R.009 for EC135 by the European Aviation Safety Agency [11]. During flight, the use of helmet with earmuffs is mandatory (Alpha Eagle Pilot Helmet). Some choose to use standard earplugs underneath the earmuffs in the helmet (either single-use foam or custom-made with individual fit) as a second ear-protective device. A total of six physicians and six pilots from the HEMS were included in the study.

### Controls

To control for hearing loss associated with working in a MECU or in the HEMS, noise exposure during daily in-hospital activities in operating theatres and in intensive care units, and DPOAE measurements were obtained in ten in-hospital anesthesiologists. These in-hospital controls were not active in the pre-hospital setting. All subjects in the control group underwent a standardized pure-tone audiometry test.

### Personal noise exposure assessment

The sound pressure level (SPL) measurements were obtained during periods of 8 h (MECU) and 12 h (HEMS) with two portable miniature microphones (DPA-4063) connected to a DPA-MPS6030 battery power supply and an Olympus LS-10 stereo digital recorder. The microphones were worn by the MECU and in-hospital anesthesiologists just lateral to the auditory canal with two custom-made holders, while the HEMS physicians and pilots had the microphones placed within the ear muffs installed in the helmets. This setup made it possible to record the actual noise reaching the ear (e.g. helicopter-generated noise or noise from the intercom communication system installed in the ear muffs of the helmets). The recordings were sampled at 44.1 kHz/16 bit in uncompressed wav format. The microphones were calibrated by recording a 1 kHz reference tone of 94 dB SPL from a B&K type 4231 sound calibrator by using a custom-made adapter to fit the microphones tight to the calibrator. Each file was reviewed through Audacity version 2.11 in order to manually check peaks and to exclude noise artefacts (e.g. unintended contact with the microphones). Finally, all recorded files were analyzed using customized MATLAB software to calculate the equivalent sound pressure level (LAeq) for the measurement period and the peak pressure level in dB(C). This setup has previously been described and validated [12].

### Types of exposures

In order to analyze the noise exposure, the MECU sound exposure measurements were separated into groups depending on their nature; that is, whether the emergency response took place on urban roads, rural roads or motorways as well as whether the measurement was obtained in the MECU en route to the patient or in the ambulance treatment compartment during transportation to the emergency department. Furthermore, it was noted whether or not the MECU physician used cellphone or handheld radio for communication. The HEMS noise exposure measurements were only separated into a physician and a pilot group, and it was noted if the HEMS physician or pilot used standard ear plugs (either single-use foam or custom-made earplugs) underneath the ear muffs installed in the helmet.

### Distortion Product Otoacoustic Emissions and pure-tone audiometry

As a baseline characteristic, each physician underwent measurements of the DPOAE amplitudes using a Titan Otoacoustic Emission Reader (Interacoustics, Middelfart, Denmark), at the beginning of a shift. Each pre-hospital physician underwent a new DPOAE test measuring the amplitude of distortion products (2f2-f1 with a f2/f1 ratio of 1.22) at 2, 3 and 4 kHz after every episode of noise exposure (either after the use of sirens on the MECU or after a flight mission with the HEMS) to calculate the level shift of the DPOAE amplitudes between baseline and post-exposure values. Only baseline values exceeding a signal to noise ratio (S/N) of 7 dB were used in the analyses. The level of the two primary DPOAE tones was L1 = 65 dB(SPL) and L2 = 55 dB(SPL). DPOAE averaging continued until the S/N of 7 dB or higher was achieved or until a maximum of 30 s (to obtain each response) had passed if the S/N of 7 dB was not achieved. All DPOAE measurements (except baseline values) were done in a non-quiet pre-hospital setting. A standard previously validated user-operated [13] pure-tone audiometry test using a computer (Compaq nx6310) connected to a Tucker Davis, Mobile digital sound processer (TDT-RM2), with Sennheiser HDA-200 headphones in a quiet room was used to reveal any hearing loss that could be ascribed to the employees in the pre-hospital setting when compared to the in-hospital controls [13]. For all participants, sex, age and additional noisy employments or hobbies (e.g. conscription to military service, hunting etc.) were recorded to rule out confounding factors.

### Statistics

The Sound Pressure Levels (SPL) of all noise exposure recordings were used as outcome variable in a linear mixed-effects regression model (xtmixed) in STATA version 14 (Stata Corp., Boca Raton, TX, USA) and the different types of exposure, measurement side (left/right) and the use of ear protection (yes/no) as fixed effects in order to obtain average equivalent sound exposures (LAT) for the different exposure types. Individuals were used as random effects in these analyses. To analyze the DPOAE data, the exposure groups were collapsed into four groups: 1) combining all MECU recordings 2) combining all ambulance recordings 3) combining all HEMS recordings and 4) the in-hospital group. The reason for collapsing the exposure groups was due to a low number of individuals with DPOAE response exceeding S/N of 7 dB at baseline in some of the exposure groups. Both MECU and ambulance DPOAE recordings were done on MECU personnel, while HEMS and in-hospital DPOAE recordings were done on HEMS personnel and controls, respectively. DPOAE amplitude level shifts between pre- and post noise exposure measurements were used as outcome variable in a mixed-effects model (xtmixed). Quality adjustments of the measured DPOAEs by inclusion of the measured noise floor and the DPOAE amplitude were included as fixed effects in the model as well as a quality check (yes/no) of DPOAE amplitudes exceeding S/N of 7 dB. Individuals were included as random effects in order to account for correlated measurements from the same individuals. To analyze the pure-tone audiometry tests, a pure-tone average (PTA) was used as outcome variable. PTA in this study is the average of the noise-sensitive hearing thresholds of the frequencies 3, 4 and 6 kHz. The same mixed-effects regression model (xtmixed) was used with PTA as outcome variable and the exposure groups including the low exposed in-hospital controls, age and sex as fixed effects to account for the known influence of age and sex on audiometric thresholds. Individuals were included as random effects. To test for overall significance of the different models, Wald χ^2^ was used.

### Legislative approval of the study

The study was approved by the Danish Data Protection Agency (2008–58-0035, journal id: 15/49437).

## Results

The investigation resulted in a total of 444 sound measurements performed during emergency responses (corresponding to 222 emergency responses) with the three MECUs in the sampling period. Each MECU physician was measured one to 34 times in total. The sound measurements in the HEMS were performed on pilots and physicians only. Each HEMS physician or pilot was measured 5–44 times, and the investigation resulted in a total of 414 sound exposure measurements. Finally, each member of the in-hospital control group was measured 1-4 times. The in-hospital investigations resulted in a total of 66 sound exposure measurements (33 for each ear). Furthermore, 408 post-exposure DPOAE measurements in total for the MECU physicians and 138 for the HEMS personnel (pilots and physicians) were obtained. Identical measurements were performed in in-hospital controls every two hours, in total collecting 32 DPOAE measurements. Finally, 12 pre-hospital physicians, 39 EMTs and 10 in-hospital controls underwent a standardized pure-tone audiometry test.

Table 1 lists the average A-weighted noise levels (L_Aeq_) throughout the entire measurement period with all the different types of exposures. As seen in Table 1, noise exposure rises with increasing speed for the MECU going from 72 dB(A) when driving in an urban environment to 75 dB(A) and 77 dB(A) when driving on rural roads and motorways, respectively. When the physician escorts the patient to the hospital in the ambulance, exposure values of 79 dB(A), 80 dB(A) and 80 dB(A) are measured. Despite the use of hearing-protective equipment, HEMS physicians and pilots are exposed to 82 dB(A) and 83 dB(A) during flight missions.

**Table 1:** Average L_Aeq_ levels and peak SPL in dB(C) levels under different types of exposure. Number of measurements in the last column

Type of exposure (Average)	Left and right ear combined (dB(A)) (Average)				Left and right ear combined (dB(C)) (Peaks)				Number of measurements (total: left + right)
	Max.	Min.	Mean	SD	Max.	Min.	Mean	SD	
Controls	83	63	69	4	122	107	113	4	66
MECU urban road	97	65	72	5	124	90	103	5	174
MECU rural road	90	67	75	4	123	99	107	4	118
MECU motorway	97	71	77	4	125	101	110	5	76
Ambulance urban road	88	73	79	4	123	100	108	6	26
Ambulance rural road	94	74	80	4	123	105	112	5	30
Ambulance motorway	85	76	80	3	122	108	114	5	20
HEMS physicians	97	73	82	5	128	109	118	4	200
HEMS pilots	97	72	83	7	128	108	118	5	214

Table 1 also lists the measured range of peak values during the different types of exposure. The largest noise exposure levels were obtained during HEMS operations with peak values reaching up to 128 dB(C) for both pilots and physicians. Again, a linear correlation is seen in the MECU group with increasing peak values as the speed of the vehicle increased.

**Fig. 1:**
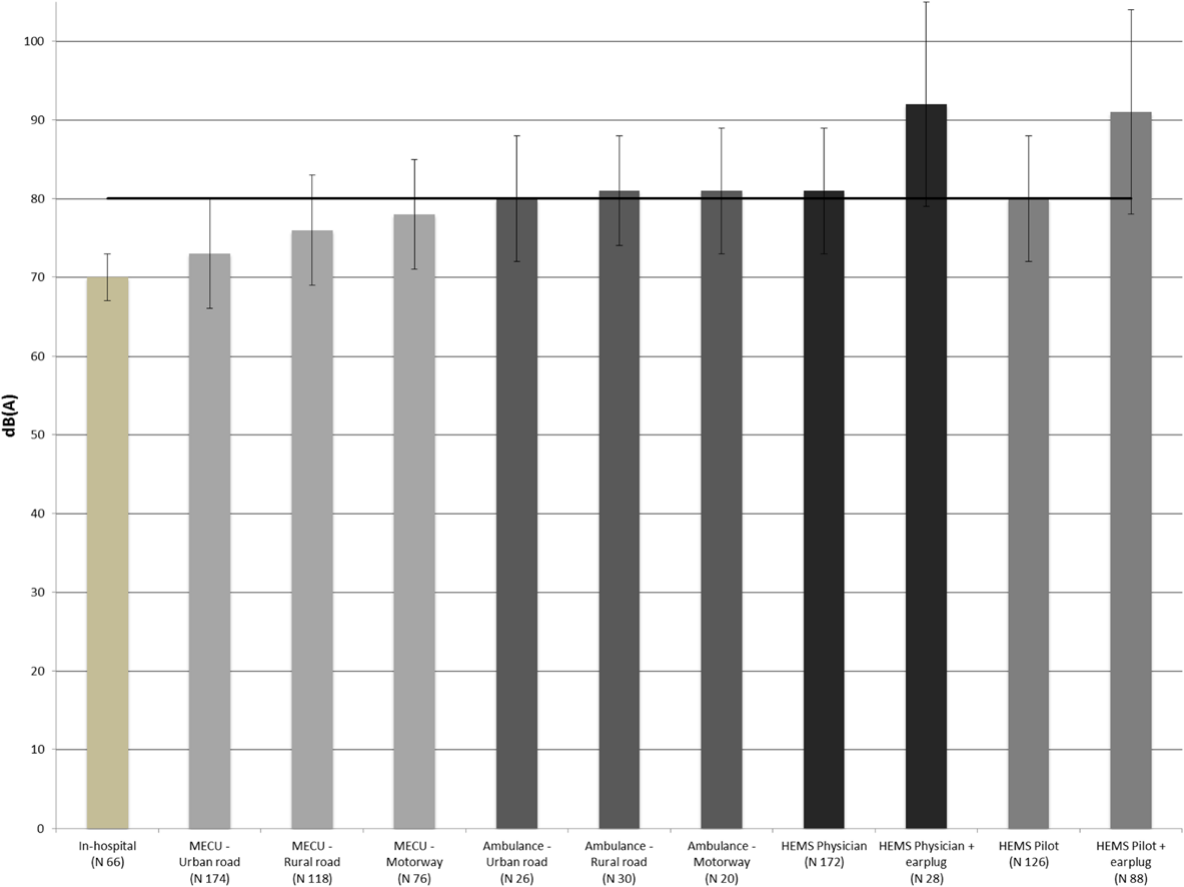
Coefficients for Noise exposure levels from the linear mixed model with “in-hospital” as reference. Error bars indicating 95% confidence interval. All exposure groups were significant larger than in-hospital controls. The horizontal bar at 80 dB represents the acceptable threshold for occupational noise as defined in the E.U. Regulative for Noise

Figure 1 shows the coefficients obtained from the linear mixed-effects regression model. All coefficients were analyzed by comparison with a reference situation, defined as the exposure of physicians working in an in-hospital environment. The linear mixed-effects regression model uses all obtained measurements and predicts the most probable noise exposure for the different exposure situations (such as MECU on rural road, MECU on motorway etc.). The exposure-specific coefficients in Fig. 1 obtained from the statistical analysis with the linear mixed-effects regression model were shown to be very similar to the mean values of the measurements in Table 1. The different types of exposure were all significantly larger than the reference coefficient (in-hospital controls). Especially interesting is the finding of the coefficients for HEMS physicians and pilots flying with earplugs, which is shown to be 11 dB(A) larger than personnel flying without standard earplugs (single-use foam or custom-made earplugs). A comparison of the noise exposure for the three MECUs demonstrated no significant difference between the three vehicles. Neither did we find any differences in the ambulance recordings.

Table 2 shows the average exposure time per emergency response during different types of exposure. On average, the MECU physicians were exposed to siren noise 42 min per day. Thirty-nine percent of the time, the exposure was during urban operations in the MECU vehicle whereas noise exposure from ambulance sirens during motorway operations contributed to 5% of the entire noise exposure. Thus, a daily noise exposure of L_eq8h_ (time-weighted average noise exposure during 8 h) of 71 dB(A) can be calculated for MECU personnel when time with non-siren exposure is taken into account. It is estimated that MECU personnel are exposed to similar noise levels as the in-hospital controls during periods outside the vehicles. HEMS personnel were on average noise-exposed to helicopter noise for 41 to 44 min during each working shift. This accounts for a daily exposure of L_eq8h_ of 73 dB(A) for both HEMS physicians and pilots when the 12-h shift is taken into account. On average, we found 5.4 emergency responses per day of 8- or 12-h recordings (with approximately one of these in the ambulance back to the hospital) with MECU and two emergency flights with HEMS.

**Table 2:** Mean exposure time per emergency response in first column inclusive 95% CI. Second column shows the total number of emergency responses during the data-collection (41 days for MECU and ambulances and 16 days for HEMS) with percentages out of total emergency responses recorded. Last column (to the right) shows the daily (8-h and 12-h with MECU and HEMS, respectively) average exposure in minutes

Type of exposure	Exposure time per emergency response (mean in minutes) [95% CI]	Total number of emergency response [% of total]	Daily average of exposure (minutes)
MECU urban road	4.6 [3.7;5.6]	87 [39%]	9.7
MECU rural road	8.2 [7.2;9.2]	59 [26%]	11.5
MECU motorway	11 [9.6;12.6]	38 [17%]	10.1
Ambulance urban road	5.8 [4;7.6]	13 [6%]	1.9
Ambulance rural road	10.5 [8.3;12.8]	15 [7%]	3.9
Ambulance motorway	18.8 [10.1;27.5]	10 [5%]	5.1
HEMS physicians	20.3 [18;22.6]	33 [100%]	41.9
HEMS pilots	20.5 [18.3;22.8]	35 [100%]	44,8

Table 3 lists the average DPOAE amplitudes (difference between baseline and post-exposure values) during different types of exposure (e.g. emergency responses with MECU, ambulance, HEMS flight missions or in-hospital control). Only the DPOAE amplitudes at 4 kHz for HEMS Crew compared to MECU measurements were significantly lower (marked in Table 3) with a *p*-value of 0.03 indicating an impact on sensory OHC function, and potential also hearing, after flight missions. None of the other measurements were statistically significantly different compared to the reference used.

**Table 3:** All measurements with MECU exposure as reference. Data are adjusted for noise floor and DPOAE amplitude signal. All included participants had recordable emissions exceeding S/N of 7 dB at baseline. The listed values are changes (Δ) from baseline values. *P* < 0.05 in bold

	Δ dB response at 2 kHz [95% CI]	Δ dB response at 3 kHz [95% CI]	Δ dB response at 4 kHz [95% CI]	Number of measurements (per 2, 3 4 kHz)
MECU exposure [reference]				
Ambulance exposure	−6 [−10;-3]	0 [−6;0]	0 [−5;4]	106 (39, 39, 28)
HEMS exposure	−6 [−13;0]	−3 [−13;2]	**−7 [−17;1]**	77 (39, 19, 19)
In-hospital exposure	4 [−1;10]	1 [−11;7]	5 [−5;13]	52 (31, 6, 15)

Pure-tone audiometry (Table 4) and the PTA did not differ between the groups when the effects of age and sex were accounted for in the analysis. However, age and sex were significant factors as expected since younger subjects had lower hearing thresholds compared to older subjects and female subjects had lower hearing thresholds compared to male subjects.

**Table 4:** PTA (3, 4 and 6 kHz combined) hearing thresholds with MECU physicians as reference group. PTA adjusted for sex and age. No pure-tone audiometry was done in HEMS physicians

	PTA hearing threshold (dB) [95% CI]	*P*-values	Mean PTA hearing threshold (dB)	Number of measurements	Active service years
Sex (coefficient) ref. female	5 [0;10]	0.05			
Age (years)	1 [0;1]	0.001			
MECU Physicians	Ref.		24	12	10
In-hospital controls	1 [−2;9]	0.27	15	10	15
EMTs	3 [−5;6]	0.81	17	39	13

## Discussion

The primary findings of this study indicate that the overall pre-hospital noise exposure during single emergency responses with MECUs and ambulances exceeds the threshold defined in the E.U. Regulative for Noise (>80 dB(A)). In single flight missions, the noise exposure with the HEMS also exceeds the threshold with exposures reaching up to 90 dB(A). However, corrected for the collective noise exposure during a whole working day, the average exposure is expected to be below the threshold for daily noise exposure. Physicians in MECU and HEMS were on average exposed to (of L_eq8h_) 71 dB(A) and 73 dB(A), respectively, and 73 dB(A) in pilots. The low average noise exposure may be the main reason why no evidence of occupational hearing loss with pure-tone audiometry could be found. In a small sample, a level change of DPOAE amplitudes for HEMS personnel at 4 kHz indicated, however, that sensory OHC function was affected and this may have a potential impact on hearing thresholds if this exposure is frequent enough.

To our knowledge, this is the first large study with systematical and simultaneous measurements of left and right ear noise exposures to pre-hospital physicians in the MECU and civilian HEMS. The largest previous study [6] only included 31 measurements in ambulances compared to our 444 MECU and ambulance recordings during different tasks (emergency response on urban roads, rural roads or motorways). Furthermore, our method using personal noise recording microphones worn just outside the auditory canal was an exact exposure model which is comparable to dosimeter use. As stated, helicopter noise exposure during alpine rescue missions has been thoroughly investigated. However, our new approach with microphones worn underneath the ear muffs in the protective helmets gives a more precise insight into the noise actually reaching the ear instead of measurements in the helicopter cabin [7]. The recorded values of 79 to 80 dB(A) in ambulances are partly in agreement with previous studies (84–96 dB(A)) with a slightly lower overall noise exposure during the use of sirens [5, 6]. A possible explanation for this is an increased focus on work-related environmental noise exposure resulting in a more appropriate siren placement and better acoustic insulation in ambulances. Despite these efforts, with maximum values reaching 94 dB(A) during ambulance transport on rural roads, noise exposure still exceeds acceptable levels and, according to the occupational guidelines, ear-protective gear must be present in the ambulances. According the E.U. regulation, these recommendations apply even if average noise exposures are below acceptable noise limits for the whole working day. The use of ear-protective gear in ambulances might impair communication with the patient during the transport to the hospital. One possible solution to this problem could be to install intercom systems to be used during transport by physicians, EMTs and the patient or to further improve the acoustic insulation of the ambulances. The noise exposure measurements in the MECU on average do not exceed the occupational noise exposure limits with measured values ranging from 72 to 77 dB(A). Semi-high levels of noise are generated when driving on motorways, probably because of the increased speed, increasing the amount of wind-generated noise. HEMS personnel not using earplugs underneath the earmuffs are exposed to average noise levels up to 81 dB(A) - and 92 dB(A) if earplugs were used.

This difference is likely caused by an impaired ability to hear the intercom when using earplugs, causing HEMS personnel to turn up the volume. Often, single-use foam earplugs were used instead of custom-made ones. Single-use foam earplugs can be expected to attenuate noise with approximately 10–20 dB(A) or less, because of variable fit to the individual auditory canal [14]. As a consequence, the protective effect of single-use foam earplugs is levelled out by increased intercom volume. Non-earplug users are still exposed above the >80 dB(A) threshold for acceptable noise according to the European Occupational Regulative [2] despite helmet usage. Therefore, further steps should be taken to reduce the occupational noise exposure to HEMS personnel. A possible intervention could be implementation of active noise reduction in all helmets. This is a well-tested initiative that for fighter pilots with similar exposure can reduce noise by 8 dB(A) [15]. Another solution could be so-called in-ears earplugs, which today are used primarily by musicians [16]. In-ears are custom-made earplugs with the possibility of radio-connection directly, and they can be used underneath the earmuffs. The advantage of in-ear plugs is that a direct connection to the intercom is possible. With the intercom inserted in the auditory canal, low volume levels can be used for communication. Overall, our findings are in accordance with earlier studies [7] despite our noise measurements in general being lower than reported (>85 dB) [7]. This difference is likely caused by the differences in methods. DPOAE post-exposure measurements were only affected for the HEMS group (Table 3) at 4 kHz when compared to MECU physicians. For both MECU and HEMS personnel, it is important to notice that the actual exposure time is limited to 42 and 41–44 min, respectively, during 8 or 12 h of recording. This limits the average daily noise exposure to L_eq8h_ of 71 dB(A) for MECU and 73 dB(A) for HEMS physicians. Both groups normally have shifts that last up to 24 h. For 24-h shifts for MECU, the average exposure will be increased by 4.8 dB compared to 8-h shifts and the increase for HEMS is 3 dB if compared to the 12-h shifts. The large distribution in 95% CI for emergency responses in ambulance on motorway is due to the geographical location of the three MECUs. Especially the MECU in Kolding will have to escort a number of patients to a larger hospital (Odense University Hospital) with up to 45 min of response time on motorway. The exposure on a particular working day therefore depends on the type of exposure during that particular day.

The DPOAEs exceeding 7 dB S/N at baseline for analysis in the HEMS group at 4 kHz Hz is quite small (*N* = 19) and the result might therefore just be an incidental finding. There are limitations to the DPOAE measurements; a person with impaired hearing will have affected emissions at baseline which may or may not exceed 7 dB S/N. Therefore, an additional affection of the DPOAE signal due to noise exposure may decrease the amplitude further, resulting in DPOAE amplitudes not exceeding 7 dB S/N. Secondly, the DPOAE equipment is susceptible to surrounding noise, which is unavoidable in a pre-hospital setting.

The pure-tone audiometry test revealed no significant differences between the three groups. This might be because of our cohort size, as a previous study of a group of EMTs revealed an occupational hearing loss [5]. Reviewing all audiograms manually, no noise-induced hearing loss could be found in any of the 61 cases. Normally, a notch is seen in the 3–6 kHz area typically centered around the 4 kHz in noise-induced hearing loss [17]. In general, pre-hospital physicians attend the MECUs and HEMS between 3 and 6 times every month, while their remaining working hours is spent within the hospitals, where the noise exposure is significantly less. This little exposure to pre-hospital noise is not expected to affect the hearing and furthermore, the exact time exposed for sirens is low. This may be an explanation why no occupational hearing loss could be found in our small cross-sectional group (with an average of 10 active pre-hospital years). Besides the risk of an occupational hearing loss, the possibility of communication problems in both MECU and HEMS settings are present due to high noise exposure. This could affect the co-operation between pre-hospital physicians and EMTs.

### Limitations

Our study was carried out on four different physician manned units in a Danish prehospital setting. Both the noise exposure recordings and DPOAE measurements were done on physicians only which is a limitation to our study. The noise recorded during MECU emergency responses is believed to reflect actual noise exposure during these types of emergency responses and is comparable at least on a national basis since similar cars and sirens used nationwide. The ambulance recordings were done only when the patient was escorted by the physician and therefore reflects noise exposure in the treatment compartment during the use of siren. It is possible that the noise exposure in the driver’s cabin is different than those recorded in the treatment compartment however; other personnel working in the treatment compartment during the use of sirens will most likely be exposed to similar noise levels as the physician. Secondly, different types of ambulances are used in Denmark which makes a direct translation of the results difficult. Worth noting, we did not find any significant differences in the recordings though several different types of ambulances were included. The HEMS recordings were done both for pilots and physicians in a way that accurately reflects the actual noise level. To generalize these results to other services require that the same helmets and earmuffs are used. In general we recognize that noise results like these can be difficult to generalize to different prehospital settings, however we believe they will help increase focus on noise being a potential occupational hazard in the prehospital emergency medical settings. Moreover it has previously been speculated that the position of microphones close to the body can cause reflection artifacts causing errors in measurements [12]. However, the method has been validated to be as accurate as using a dosimeter placed according to ISO 9612 with the generally accepted 2 dB measurement uncertainty [12]. Another potential weakness of our study is the possibility of conduction of noise through the body (bone conduction) because of vibrations (especially in the HEMS) since the measurements in this study reflect the air-conducted noise only. As with every study, the risk of recruitment bias is present; i.e. if a person with a known hearing loss rejects enrollment to the project, the possibility of detecting an overall hearing loss is less if all subjects are known to have a good hearing.

## Conclusion

Our study indicates that the overall pre-hospital noise exposure during emergency responses with MECUs, ambulances and HEMS missions exceeds the threshold defined in the E.U. Regulative for Noise (>80 dB(A)). Although no evidence of occupational hearing loss with pure-tone audiometry could be established and, in general, average noise exposure during the whole working day was low, we recommend either better insulation of MECUs and ambulances, or the opportunity to use ear-protective gear such as ear muffs or earplugs. Furthermore, ear-protective gear should likewise be offered to HEMS personnel as they are heavily exposed to noise despite the passive ear-protective gear already in use.

## Abbreviations

DPOAE: Distortion Product Otoacoustic Emissions
EMT: Emergency Medical Technician
HCM: HEMS Crew Member
HEMS: Helicopter Emergency Medical Services
LAeq: Equivalent Sound Pressure Limit
LAT: Equivalent Sound Pressure
MECU: Mobile Emergency Care Unit
OHC: Outer Hair Cells
PTA: Pure Tone Average
S/N: Signal to noise ratio
SPL: Sound Pressure Level
TTS: Temporary Threshold Shift
